# Impact of an unrestricted clear liquid fasting protocol on pediatric perioperative outcomes: a prospective interventional study

**DOI:** 10.1007/s00383-026-06558-5

**Published:** 2026-08-03

**Authors:** Alexander S. Mina, Dimingo Gomez, Duc T. Nguyen, J. Matthew Kynes, Jason Axt

**Affiliations:** 1 Department of Surgery, Baylor College of Medicine, 1 Baylor Plaza, Houston, TX 77030 USA; 2 Division of Pediatric Surgery, AIC Kijabe Hospital, 00220 Kijabe, Kenya

**Keywords:** Preoperative fasting, Clear liquids, Pediatric surgery, Patient satisfaction, Quality improvement

## Abstract

**Purpose:**

Traditional preoperative fasting guidance (6-4-2) aims to reduce aspiration risk but frequently produces prolonged fasting and discomfort in children. We evaluated whether progressively liberalizing the clear-liquid fasting policy reduces fasting time and improves the perioperative experience.

**Methods:**

We conducted a prospective, four-phase interventional quality-improvement study at a tertiary hospital in rural Kenya (11/2021-12/2022). Phase 0 followed standard 6-4-2 guidelines; Phase 1 shortened clear-liquid fasting to 1 h; Phase 2 permitted a fixed volume of water until transfer to theatre; Phase 3, unrestricted water. The primary outcome was clear-liquid fasting time. Secondary outcomes were thirst, hunger, parental satisfaction, preoperative anxiety, and aspiration events.

**Results:**

Among 253 children, median clear-liquid fasting time decreased: 14.0 h (Phase 0) to 1.7 h (Phase 3) (*p* < 0.001). Anxiety decreased significantly (mYPAS 35.4 to 22.9; *p* < 0.001) and thirst declined. Parental satisfaction was higher but did not reach statistical significance (8.0 to 9.0; *p* = 0.06). One confirmed aspiration occurred in Phase 1 (7.2-h fasting time).

**Conclusion:**

Reducing preoperative fasting improved comfort and lowered anxiety. No increase in clinically significant aspiration events was observed, although the study was not powered for rare events. An unrestricted clear-liquid protocol appears feasible and beneficial.

**Supplementary Information:**

The online version contains supplementary material available at 10.1007/s00383-026-06558-5.

## Introduction

Traditional guidance for preoperative pediatric fasting has recommended at least 6 h for solids or non-human milk, 4 h for breast milk, and 2 h for clear liquids (the 6-4-2 rule), with the aim of reducing the risk of pulmonary aspiration of gastric contents under anesthesia. The 2-h clear-liquid rule has increasingly been challenged on practical, physiological, and psychological grounds. Gastric-emptying studies using ultrasound and magnetic resonance imaging demonstrate that water is reliably cleared from the stomach within approximately 30 min [1], and several national anesthesia societies have shortened their recommended clear-fluid fasting interval from 2 h to 1 h [2–4]. Some institutions have gone further, permitting clear liquids until the time of surgery (a 0-h policy) [5]. Reported experience with these more liberal policies, however, comes largely from observational and quality-improvement work, and direct comparisons of 1-h and 0-h approaches remain sparse.

In practice, children frequently fast far longer than guidelines intend, with average clear-liquid fasting times of 6–9 h and fewer than 20% of children achieving less than 4 h [6, 7]. Caregiver reluctance to give fluids for fear of cancellation, unpredictable theatre scheduling, and emergency cases all contribute to prolonged fasting. Prolonged fasting carries measurable consequences: in a study of approximately 15,000 patients, those who fasted 4 h or more had a 30% higher risk of hypotension after induction than those who fasted less than 4 h [8]; prolonged fasting is also associated with increased insulin resistance, ketogenesis, and hypoglycemia [9], and children report substantial hunger and thirst [10]. Quality-improvement programs that shortened clear-fluid fasting from 2 h to 1 h have reduced prolonged fasting by more than 50% [6, 7], and liberalized policies have been associated with shorter fasting times and improved patient well-being without a detected increase in aspiration [11–13].

Despite this growing evidence, adoption of unrestricted clear-fluid protocols remains variable, and few data describe their implementation in low-resource settings. AIC Kijabe Hospital is a tertiary mission hospital in rural Kenya that cares for approximately 2,000 pediatric surgical patients annually, where stakeholders suspected that preoperative fasting times were prolonged. This study describes the impact of a phased reduction in clear-liquid fasting restrictions on fasting time, patient and caregiver experience, and observed safety events. We hypothesized that progressively liberalizing the clear-liquid fasting policy would substantially reduce fasting time and improve the perioperative experience, without an observed increase in clinically significant aspiration events.

## Methods

### Study design and setting

This was a prospective, single-center, interventional study conducted as a quality-improvement initiative at AIC Kijabe Hospital, a tertiary mission hospital serving a predominantly rural population in Kenya. The study combined a baseline observational phase with three sequential interventional phases, each representing a stepwise liberalization of the preoperative clear-liquid fasting policy, and was carried out between November 2021 and December 2022. Reporting follows the Standards for Quality Improvement Reporting Excellence (SQUIRE 2.0) where applicable.

We included all patient encounters in which a child underwent an elective surgical procedure performed by the pediatric surgery service during the study period. Eligible patients were between one month and 18 years of age on the day of surgery and were scheduled as outpatient or same-day-admission cases. We excluded patients undergoing emergency surgery; those with obstructive esophageal pathology (e.g., achalasia, esophageal stricture), known delayed gastric emptying, or bowel obstruction; and those with incomplete fasting-time data.

### Study phases

The previously existing institutional policy followed standard 6-4-2 fasting (at least 6 h for solids and non-human milk, 4 h for breast milk, and 2 h for clear liquids). In each of the three interventional phases, breast-milk fasting was shortened to 3 h and the clear-liquid policy was modified as described below.

**Phase 0 (baseline).** Observational phase; patients followed the existing 6-4-2 policy, with a 2-h clear-liquid fast.

**Phase 1.** Clear-liquid fasting was reduced to 1 h (6-3-1).

**Phase 2.** A 0-h clear-liquid policy with a limited volume was introduced (6-3-0). Children were permitted water up to the time of entering the operating room; a designated staff member at the theatre reception offered water at hourly intervals using calibrated cups − 30 mL for children younger than 3 years, 60 mL for those aged 3–8 years, and 120 mL for those older than 8 years.

**Phase 3.** The volume limit was removed; children were permitted unrestricted water until being called to theatre (6-3-0, unrestricted).

### Outcomes

The primary outcome was total clear-liquid fasting time, measured as the interval between the last reported or observed ingestion of clear liquids and induction of anesthesia. Secondary outcomes were perioperative regurgitation (gastric contents in the upper airway during induction or emergence), suspected perioperative aspiration (coughing or laryngospasm associated with regurgitation), confirmed aspiration (gastric contents in the lower airway or endotracheal tube during induction or emergence), patient-reported hunger and thirst (yes/no), parental satisfaction rated preoperatively on a 0–10 scale, and preoperative anxiety measured with the Modified Yale Preoperative Anxiety Scale (mYPAS) [14] before the procedure and on arrival to the operating room.

### Data collection and management

Designated data collectors recorded patient demographics, fasting times, and outcomes on standardized paper forms; parental satisfaction was assessed using a visual analog scale. Data were subsequently entered into a password-protected Research Electronic Data Capture (REDCap) database.

### Statistical analysis

Descriptive data are presented as counts and proportions, or as medians with interquartile ranges (IQR) for non-normally distributed continuous variables. As a pragmatic quality-improvement study, no formal a priori power calculation was performed for rare adverse events such as aspiration; rather, a target enrolment of approximately 50–60 patients per phase was set to detect clinically meaningful reductions in fasting time and to permit assessment of secondary outcomes using non-parametric methods. The primary outcome and other continuous outcomes were compared across phases using the Kruskal–Wallis test, and categorical outcomes using the chi-square or Fisher’s exact test, as appropriate for cell counts. To estimate the adjusted association between study phase and clear-liquid fasting time, a generalized linear model (GLM) with a Gaussian family and log link was fitted, adjusting for age and sex; results are reported as exponentiated coefficients representing the relative change in expected fasting time, with pairwise comparisons against Phase 0. A two-sided p value < 0.05 was considered statistically significant. Analyses were performed in Stata (StataCorp, College Station, TX, USA).

### Ethical considerations

The study was approved by the AIC Kijabe Hospital Institutional Scientific and Ethics Review Committee and was performed in accordance with the principles of the Declaration of Helsinki. Written informed consent was obtained from the parent or legal guardian of every participating child before enrolment; consent documents were available in both English and Kiswahili. A predefined safety plan provided for review of every episode of regurgitation or aspiration during the study to identify contributing factors. As a single-institution quality-improvement initiative modifying an element of routine perioperative care, the study was not registered in a clinical-trials registry.

## Results

### Patient characteristics

During the study period, 282 patients were screened. After exclusion of 29 patients with missing clear-liquid fasting times, 253 patients were included in the analysis: 55 in Phase 0, 53 in Phase 1, 54 in Phase 2, and 91 in Phase 3. The median age of the cohort was 52.4 months (IQR 26.8–90.3), with no significant difference across phases (*p* = 0.64), and most patients were male (77.5%; *p* = 0.36 across phases). General anesthesia with an endotracheal tube or laryngeal mask was used in 95% of cases, and facemask sedation in 5%. Urology procedures were the most common operation type overall (52.2%). Patient and procedural characteristics are summarized in Table 1.

**Table 1:** Patient and procedural characteristics

Characteristic	Phase 0 (*n* = 55)	Phase 1 (*n* = 53)	Phase 2 (*n* = 54)	Phase 3 (*n* = 91)	*p* value
Age (months), median (IQR)	53.8 (35.9–89.7)	56.7 (28.3–84.9)	50.8 (20.8–86.6)	52.4 (22.6–105.1)	0.64
*Sex, n (%)*					0.36
Male	39 (71)	39 (74)	43 (80)	75 (82)	
Female	16 (29)	14 (26)	11 (20)	16 (18)	
*ASA class, n (%)* ^a^					< 0.001*
I	6 (11)	17 (35)	8 (15)	48 (53)	
II	48 (87)	30 (63)	45 (83)	42 (47)	
III	1 (2)	1 (2)	1 (2)	0 (0)	
*Operation type, n (%)* ^b^					0.008*
Urology	27 (49)	27 (51)	27 (50)	50 (55)	
Gastrointestinal	19 (35)	16 (30)	14 (26)	14 (15)	
Skin/soft tissue	5 (9)	5 (9)	12 (22)	24 (26)	
Other	4 (7)	5 (9)	0 (0)	2 (2)	
*Admission status, n (%)* ^c^					< 0.001*
Day case	47 (85)	42 (79)	38 (72)	90 (99)	
Admitted	8 (15)	11 (21)	15 (28)	1 (1)	

ASA American Society of Anesthesiologists physical status, IQR interquartile range. p values are from the Kruskal–Wallis test (age) or the Chi-square test (categorical variables) and compare all four phases. *Statistically significant differences reflect the case-mix shift across phases: Phase 3 included a higher proportion of ASA class I patients, fewer gastrointestinal and more skin/soft-tissue procedures, and a higher proportion of day cases; see Patient and procedural characteristics in the Results for details

^a^ASA class was missing for 6 patients (5 in Phase 1, 1 in Phase 3)

^b^Operation type was missing for 2 patients (1 each in Phases 2 and 3)

^c^Admission status was missing for 1 patient (Phase 2). Percentages for these variables are calculated among patients with available data

Case mix differed across phases. ASA classification and admission status both varied significantly (*p* < 0.001 for each): Phase 3 contained a higher proportion of ASA I patients (52.7%) than Phase 0 (10.9%) and consisted almost entirely of day-case procedures (98.9% vs. 85.5% in Phase 0), with only one Phase 3 patient admitted rather than discharged on the day of surgery. Operation type also differed across phases (*p* = 0.008). These differences are considered further in the Discussion.

### Clear-liquid fasting time

**Fig. 1:**
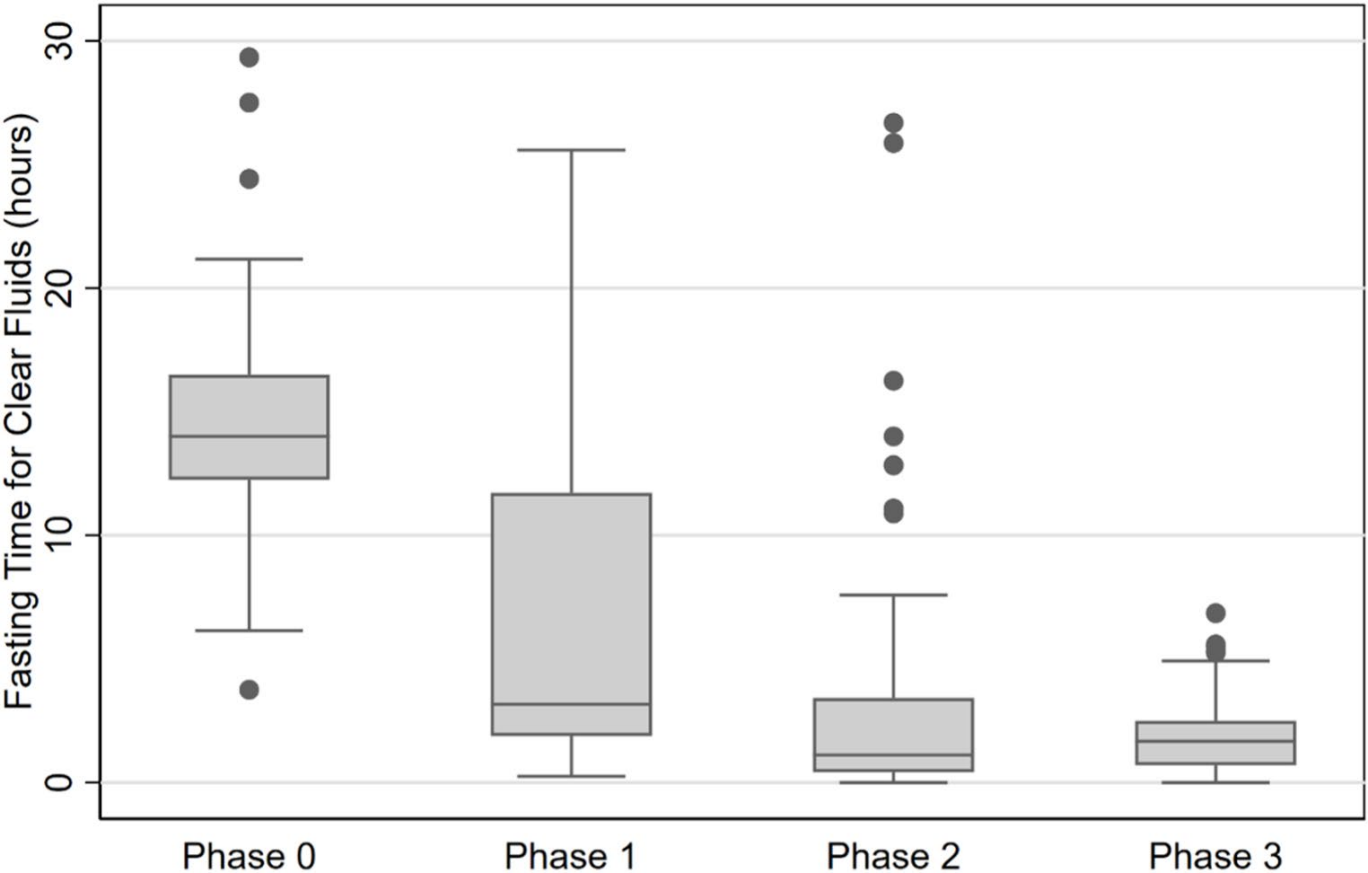
Preoperative clear-liquid fasting duration by study phase. Box plots compare the baseline observational phase (Phase 0) with the three interventional phases (Phases 1–3). Kruskal–Wallis *p* < 0.001 across phases; each interventional phase differed significantly from Phase 0

Clear-liquid fasting time fell substantially after the intervention. The median fasting time was 14.0 h (IQR 12.3–16.5) in the baseline phase, 3.2 h (IQR 1.9–11.7) in Phase 1, 1.1 h (IQR 0.4–3.4) in Phase 2, and 1.7 h (IQR 0.7–2.5) in Phase 3 (Kruskal–Wallis *p* < 0.001; Table 2; Fig. 1). In the adjusted GLM, every interventional phase was associated with a significant reduction in expected fasting time relative to Phase 0 after adjustment for age and sex (Table 3); Phase 3 was associated with an 87% relative reduction in expected clear-liquid fasting time (exponentiated coefficient 0.13, 95% CI 0.08–0.22; *p* < 0.001).

**Table 2:** Perioperative outcomes by study phase

Outcome	Phase 0 (*n* = 55)	Phase 1 (*n* = 53)	Phase 2 (*n* = 54)	Phase 3 (*n* = 91)	*p* value
Clear-liquid fasting time (h), median (IQR)	14.0 (12.3–16.5)	3.2 (1.9–11.7)	1.1 (0.4–3.4)	1.7 (0.7–2.5)	< 0.001
Parental satisfaction (0–10), median (IQR)	8.0 (7.0–10.0)	9.5 (8.0–10.0)	9.0 (8.0–10.0)	9.0 (8.0–10.0)	0.06
mYPAS anxiety score, median (IQR)	35.4 (22.9–50.0)	29.2 (27.1–35.4)	29.2 (22.9–52.1)	22.9 (22.9–37.5)	< 0.001
Reports of hunger, n (%)	29 (81)	21 (66)	28 (80)	28 (56)	0.04
Reports of thirst, n (%)	28 (76)	10 (32)	5 (14)	32 (64)	< 0.001
Intravenous cannulation attempts, median (IQR)	1 (1–2)	1 (1–2)	1 (1–2)	1 (1–2)	0.40

mYPAS Modified Yale Preoperative Anxiety Scale, IQR interquartile range. p values are global tests comparing all four phases (Kruska–Wallis test for continuous outcomes; chi-square or Fisher’s exact test for categorical outcomes) and indicate whether an outcome differed across phases overall; they do not denote specific pairwise differences. Hunger and thirst percentages are calculated among patients who responded, not the total n

**Table 3:** Demographic-adjusted association between study phase and clear-liquid fasting time (generalized linear model)

Variable	Exponentiated coefficient	95% CI	*p* value
Phase 0 (6-4-2)	Reference	–	–
Phase 1 (6-3-1)	0.48	0.39–0.58	< 0.001
Phase 2 (6-3-0, volume-limited)	0.27	0.19–0.37	< 0.001
Phase 3 (6-3-0, unrestricted)	0.13	0.08–0.22	< 0.001
Age (months)	1.001	1.000-1.002	0.101
Sex (female)	1.03	0.87–1.21	0.732

CI confidence interval. Generalized linear model (Gaussian family, log link) with clear-liquid fasting time as the outcome. Exponentiated coefficients represent the relative change in expected fasting time; a value below 1 indicates a reduction relative to Phase 0. The model was fitted on 251 patients; 2 patients were excluded for missing age data

### Patient experience: satisfaction, thirst, hunger, and anxiety

Parental satisfaction (0–10 scale) was higher in the interventional phases, with the median rising from 8.0 in Phase 0 to 9.0; this difference did not reach statistical significance (*p* = 0.06) and should not be interpreted as a true between-phase difference.

Reports of thirst fell markedly during the first two interventional phases, from 75.7% of responding patients in Phase 0 to 14.3% in Phase 2, before rebounding to 64.0% in Phase 3 (*p* < 0.001). Reports of hunger decreased from 80.6% in Phase 0 to 56.0% in Phase 3 (*p* = 0.04).

Preoperative anxiety improved across phases: the median mYPAS score decreased from 35.4 (IQR 22.9–50.0) in Phase 0 to 22.9 (IQR 22.9–37.5) in Phase 3 (*p* < 0.001).

### Safety events

Safety events were uncommon. Perioperative regurgitation occurred in five patients overall, with no significant difference across phases (Phase 0, *n* = 1; Phase 1, *n* = 2; Phase 2, *n* = 0; Phase 3, *n* = 2; Fisher’s exact *p* = 0.578). Four patients showed clinical signs of suspected aspiration, one of which met criteria for confirmed aspiration; all four occurred in the two most restrictive phases (Phase 0, *n* = 1; Phase 1, *n* = 3), and none occurred in Phase 2 or Phase 3 (Fisher’s exact *p* = 0.037). Because this comparison is based on only four events, it should be regarded as exploratory rather than as reliable evidence of a true between-phase difference. The single confirmed aspiration occurred in Phase 1, in a 56-month-old child undergoing a gastrointestinal procedure whose documented clear-liquid fasting time was 7.2 h—well beyond the 1-h protocol target. This child developed a postoperative pulmonary complication that was clinically minor and self-limited, was managed with supportive care alone, and required neither supplemental oxygen nor hospital admission. No perioperative regurgitation or aspiration event in any phase was associated with a major complication of Clavien-Dindo grade III or higher [15]. Event-level details are presented in Table 4.

**Table 4:** Detail of perioperative regurgitation and aspiration events

Phase	Age (months)	Procedure	NPO solid (h)	NPO clear (h)	Event
Phase 1	56.7	Gastrointestinal	7.2	7.2	Confirmed aspiration
Phase 0	89.0	Gastrointestinal	11.4	11.4	Suspected aspiration
Phase 1	–	Other	20.1	4.6	Suspected aspiration
Phase 1	155.2	Urology	20.4	1.4	Suspected aspiration
Phase 0	25.3	Gastrointestinal	10.8	14.3	Regurgitation
Phase 1	–	Gastrointestinal	17.8	2.8	Regurgitation
Phase 1	64.5	Urology	18.5	2.5	Regurgitation
Phase 3	9.5	Skin/soft tissue	31.5	0.5	Regurgitation
Phase 3	21.3	Skin/soft tissue	15.2	1.6	Regurgitation

NPO nil per os. Events are listed individually. The single confirmed aspiration occurred in Phase 1 in a patient whose clear-liquid fasting time (7.2 h) exceeded the protocol target; the associated postoperative pulmonary complication was clinically minor and self-limited; no event was associated with a Clavien-Dindo grade III or higher complication. Dashes indicate data not recorded

## Discussion

In this four-phase quality-improvement study, progressively liberalizing the preoperative clear-liquid fasting policy was feasible and was accompanied by a substantial reduction in clear-liquid fasting time, from a median of 14.0 h under standard 6-4-2 fasting to 1.7 h under an unrestricted-water protocol. The magnitude of this reduction is consistent with previous observational and quality-improvement reports of liberalized fasting [11–13] and was achieved through a simple, low-cost change to theatre-side workflow.

The intervention was also associated with improvements in the patient and caregiver experience. Preoperative anxiety, measured by the mYPAS, decreased significantly across phases—a clinically relevant finding, as preoperative anxiety has been linked to maladaptive postoperative behavior and increased analgesic requirements [16]. This is consistent with prior reports on the discomfort and agitation associated with prolonged preoperative fasting in children [10, 17]. Reports of thirst also fell sharply in the first two interventional phases. Assessing these outcomes in children is, however, inherently difficult: anxiety scoring is affected by the high-stress environment of the waiting and operating rooms and by the challenge of obtaining reliable reports from young children, and satisfaction and symptom reports are subjective. Parental satisfaction ratings may additionally have been influenced by the absence of a prior reference point—for most families this was their first experience of a child undergoing surgery—and by an understandable reluctance to report dissatisfaction while their child remained under the care of the surgical team. The non-significant difference in parental satisfaction (*p* = 0.06) should therefore be regarded as hypothesis-generating rather than as evidence of a true effect.

An unexpected finding was the rebound in reported thirst during Phase 3, despite this phase having among the shortest in-hospital fasting times. One plausible explanation is variability in theatre scheduling and waiting times: children who waited longer may have felt thirstier even while remaining eligible to drink. It is also important to recognize that the intervention principally addressed fasting that accrues after arrival at the hospital. Prolonged fasting that begins at home—driven by caregiver caution and early or uncertain reporting times—was not directly targeted. Preoperative communication with families, such as a reminder on the day before surgery to continue offering clear fluids, is a logical next step and may further shorten total fasting time. We did not find a direct correlation between reported hunger and clear-liquid fasting time, which is expected given that the protocol addressed clear liquids rather than solid intake.

Preoperative fasting exists primarily to reduce the risk of pulmonary aspiration, and the safety of shorter fasting therefore warrants careful interpretation. We observed one confirmed aspiration event, in a Phase 1 patient whose documented clear-liquid fasting time of 7.2 h substantially exceeded the protocol target; the associated postoperative pulmonary complication was clinically minor and self-limited, and no aspiration-related complication of Clavien-Dindo grade III or higher was observed in any phase. No confirmed or suspected aspiration events occurred in the two phases with the most liberal fasting (Phases 2 and 3). These observations are reassuring but must not be over-interpreted. Pulmonary aspiration is a rare event, with a reported incidence of roughly 1 in 10,000 anesthetics, and this study—like most single-center studies of fasting—was neither designed nor powered to detect a difference in aspiration risk. Our findings should not be read as demonstrating that an unrestricted clear-fluid policy is definitively safe. It is equally important to be precise about the existing literature: large studies such as the 6-4-0 cohort of Andersson et al. [5] and the German NiKs study [11] demonstrate that liberalized clear-fluid fasting reduces prolonged fasting and is accompanied by a low observed incidence of aspiration, but they were not themselves powered to establish the aspiration safety of a true 0-h policy. Establishing safety with confidence will require adequately powered, preferably multicenter, studies.

Our findings are also consistent with the tenets of enhanced recovery after surgery (ERAS), which emphasize minimizing preoperative fasting, maintaining hydration and metabolic homeostasis, and reducing perioperative stress and opioid requirements. Although most ERAS evidence derives from adult practice, a growing body of literature supports similar effectiveness in the pediatric population [18, 19]. In this context, the near-universal day-case rate in Phase 3 (98.9%), with only one child requiring inpatient admission, accords with the ERAS goals of adequate preoperative hydration, minimized perioperative stress, and early discharge. Because admission status in this sequential study reflected scheduling patterns and case mix as well as actual postoperative disposition, we regard this observation as supportive of, rather than attributable to, the liberalized protocol. Finally, the unrestricted clear-fluid policy required no additional equipment or personnel and has remained the institutional standard of care at our center since the completion of the study, supporting its sustainability in a low-resource setting.

### Limitations

This study has several limitations. First, as noted, the sample size was insufficient to assess the incidence of rare adverse events such as aspiration, and the safety comparisons across phases are based on very few events and should be regarded as exploratory. Second, the design was a sequential, non-randomized quality-improvement intervention, and case mix shifted across phases: later phases included a higher proportion of ASA I patients and of day-case procedures. The adjusted model for the primary outcome accounted for age and sex but not for ASA class or admission status, so residual confounding cannot be excluded; however, the very large reduction in fasting time, its consistency across all interventional phases, and its clear temporal link to each policy change make case mix an unlikely explanation for the primary finding. Third, this was a single-center study at a mission hospital in rural Kenya, which may limit generalizability to institutions with different patient populations, staffing, and resources. Fourth, some variables had missing data, including fasting times, ASA class, and anxiety scores. Finally, satisfaction and symptom outcomes were self-reported and subject to measurement limitations.

## Conclusion

In a resource-limited setting, a phased move toward an unrestricted preoperative clear-fluid policy was feasible and was associated with markedly shorter fasting times and improvements in thirst and preoperative anxiety, without an observed increase in clinically significant aspiration events. These findings are practice-informing and hypothesis-generating: they support the continued, carefully monitored implementation and evaluation of liberalized clear-fluid protocols, and they add real-world experience from a setting where such protocols have not been widely adopted. Because the intervention relied on a low-cost adjustment to theatre-side workflow rather than new equipment or infrastructure—its principal requirement being a designated staff member to offer and document clear fluids—it has proved sustainable—remaining our institutional standard since study completion—and is likely to be transferable to comparable resource-limited settings. Adequately powered, multicenter studies remain necessary to define the safety of a 0-h clear-fluid policy and to guide its broader adoption.

## Supplementary Information

Below is the link to the electronic supplementary material.

Supplementary Material 1

## Data Availability

The data that support the findings of this study are available from the corresponding author upon reasonable request.
