# Supplementary material for: Impact of an unrestricted clear liquid fasting protocol on pediatric perioperative outcomes: a prospective interventional study
Source: Pediatr Surg Int. 2026 Aug 3;42(1):328. doi: 10.1007/s00383-026-06558-5 (PMC13433598; doi:10.1007/s00383-026-06558-5)
Supplement: Supplementary file 1 [file 383_2026_6558_MOESM1_ESM.docx]

**SQUIRE 2.0 Reporting Checklist**

*Manuscript: “Impact of an Unrestricted Clear Liquid Fasting Protocol on Pediatric Perioperative Outcomes” — prepared for Pediatric Surgery International*

**Standards for Quality Improvement Reporting Excellence (SQUIRE 2.0).**

| **SQUIRE 2.0 item** | **Item description (abbreviated)** | **Location in manuscript** |
| --- | --- | --- |
| **Title and Abstract** | | |
| **1. Title** | Indicates the manuscript concerns an initiative to improve healthcare. | Title (page 1 and title page). The study is identified as a four-phase interventional quality-improvement initiative in the Abstract and in Methods – Study design and setting. |
| **2. Abstract** | Provides indexing information and summarizes key information in a structured format. | Abstract, structured as Purpose, Methods, Results, Conclusion (per the PSI format). |
| **Introduction — Why did you start?** | | |
| **3. Problem description** | Nature and significance of the local problem. | Introduction, paragraphs 2–3 (prolonged preoperative fasting suspected at AIC Kijabe Hospital, and its consequences). |
| **4. Available knowledge** | What is currently known, including relevant previous studies. | Introduction, paragraphs 1–2 (6-4-2, 1-hour and 0-hour policies; prior outcomes). |
| **5. Rationale** | Frameworks/assumptions used to develop the intervention and why it was expected to work. | Introduction, paragraph 1 (gastric-emptying physiology) and final paragraph (explicit hypothesis). |
| **6. Specific aims** | Purpose of the project and of this report. | Introduction, final paragraph (aim and hypothesis). |
| **Methods — What did you do?** | | |
| **7. Context** | Contextual elements important at the outset. | Introduction, paragraph 3; Methods – Study design and setting (tertiary mission hospital, rural Kenya, ~2,000 pediatric surgical cases/year, resource-limited). |
| **8. Intervention(s)** | Description in reproducible detail; the team involved. | Methods – Study phases (four-phase protocol; Phase 2 calibrated volumes by age; designated theatre-reception staff member; data collectors). |
| **9. Study of the intervention(s)** | Approach to assessing impact and to attributing outcomes to the intervention. | Methods – Study design and setting; Statistical analysis (baseline observational phase plus three sequential interventional phases; demographic-adjusted GLM). |
| **10. Measures** | Measures, operational definitions, validity/reliability; completeness and accuracy of data. | Methods – Outcomes (operational definitions of each outcome; validated mYPAS instrument); Data collection and management (REDCap database; missing-data handling). |
| **11. Analysis** | Methods used to draw inferences; understanding variation, including the effect of time. | Methods – Statistical analysis (Kruskal–Wallis, chi-square/Fisher, GLM; study phase analyzed as the sequential/time variable). |
| **12. Ethical considerations** | Ethical aspects, formal ethics review, and conflicts of interest. | Methods – Ethical considerations (institutional ethics-committee approval; informed consent; predefined safety-review plan); Declarations – Competing interests. |
| **Results — What did you find?** | | |
| **13. Results** | Evolution of the intervention; process and outcome measures; contextual elements; associations; unintended consequences; missing data. | Results, all subsections; Tables 1–4; Fig. 1. Includes the across-phase case-mix shift (contextual element), the Phase 3 thirst rebound (unintended consequence), and patient flow / missing-data accounting. |
| **Discussion — What does it mean?** | | |
| **14. Summary** | Key findings relative to the aims; strengths of the project. | Discussion, paragraph 1; Conclusion (feasibility and a simple, low-cost workflow change as strengths). |
| **15. Interpretation** | Nature of the association; comparison with other work; impact; reasons for unexpected results; trade-offs. | Discussion, paragraphs 1–4 (literature comparison, patient-experience and workflow impact, explanation of the thirst rebound); Conclusion (staff-time trade-off). |
| **16. Limitations** | Limits to generalizability; threats to internal validity; efforts to address them. | Discussion – Limitations (underpowered for aspiration; non-randomized sequential design; case-mix confounding; single-center generalizability; missing data). |
| **17. Conclusions** | Usefulness; sustainability; potential for spread; implications for practice and study; next steps. | Conclusion (sustainability and transferability of the low-cost workflow change; need for adequately powered multicenter studies; day-before caregiver communication as a next step). |
| **Other information** | | |
| **18. Funding** | Sources of funding and the role of any funder. | Declarations – Funding; Title page (no funding received; no funder role). |

**Reference.** Ogrinc G, Davies L, Goodman D, Batalden P, Davidoff F, Stevens D (2016) SQUIRE 2.0 (Standards for QUality Improvement Reporting Excellence): revised publication guidelines from a detailed consensus process. BMJ Qual Saf 25:986–992.
